# Study of Hole-Transporter-Free Perovskite Solar Cells based on Fully Printable Components

**DOI:** 10.3390/mi10040266

**Published:** 2019-04-20

**Authors:** Camellia Raminafshar, Dimitrios Raptis, Mohammad Reza Mohammadi, Panagiotis Lianos

**Affiliations:** 1 Department of Materials Science and Nanotechnology, School of Science and Engineering, Sharif University of Technology, International Campus-Kish Island, Kish Island 79417-76655, Iran; raminafshar@kish.sharif.ir; 2 Department of Chemical Engineering, University of Patras, 26500 Patras, Greece; dgraptis86@yahoo.gr; 3 Department of Materials Science and Engineering, Sharif University of Technology, Tehran 14588-89694, Iran

**Keywords:** perovskite solar cells, hole-transporter free, fully-printable components

## Abstract

Hole-transporter-free perovskite solar cells carrying a carbon back contact electrode provide the possibility of making full printable low cost and stable devices, even though their efficiency is substantially lower than those made in the standard configuration. The present work searched for simple and easy routes for constructing such devices, demonstrating that organic components do enhance device efficiency but only to a level that is not worth the trouble nor the cost. Devices based on a triple mesoporous layer of titania/zirconia/carbon with perovskite infiltration gave an efficiency of 10.7%. After 180 days of storing under ambient conditions, a small loss of efficiency has been observed for a cell made in June, in spite of the fact that in going from June to December, a large increase of the ambient humidity took place, thus verifying the protective effect that the carbon electrode is providing. The addition of spiro-OMeTAD to the hole-transporter-free device resulted in increasing the efficiency by about 10%, a change which is appreciated to be of low importance given the cost of this material. This increase mainly derived from an increase in the current. Devices of different sizes have been constructed by screen printing, using home-made pastes for all the components making the cell scaffold, i.e., for titania, zirconia, and carbon layers.

## 1. Introduction

Organometal halide perovskite solar cells (PSC) have established themselves as the most promising new generation solar cell technology. This took place in a rather short period of time since their first application in 2009 as sensitizer in dye sensitized solar cells [1]. Their success is attributed to their excellent optoelectronic properties and the easiness of their synthesis and application by “soft” chemistry solution processing. These properties are listed and analyzed in many review papers. We cite here a few of the recent ones [2,3,4]. Even though, the time span of PSC studies is relatively short, the large number of involved researchers and the subsequent number of publications resulted in the study of a large variety of materials and of their combinations in the construction of PSC variants. The vast majority of reported studies is based on the following standard configuration: on a fluorine-doped tin oxide (FTO) transparent electrode, a thin titania blocking layer (bl-TiO_2_) is first deposited followed by a mesoporous titania layer (m-TiO_2_), which plays the role of scaffold for the development of the active perovskite layer and the role of electron transporter from the perovskite to the FTO anode. On top of mesoporous titania, the perovskite layer is deposited by liquid phase mixing of precursor reagents. These reagents, usually an organic halide and a lead halide, interact to form the organometal halide perovskite aided by mild annealing. Finally, a solution processed layer of an organic hole transporter is added on the top and the device is completed by depositing a noble metal counter electrode. This standard configuration resulted in a certified 22.1% efficiency [5], even though, most researchers do not achieve this record score and they publish results ranging between 15 and 20% [6,7,8,9,10]. The cell efficiency increases by adding a buffer layer composed of a mesoporous inert material between the perovskite layer and the hole transporting layer. This layer protects the perovskite from the metal electrode, blocks shunt paths through the device layers and organizes the dispersion of the hole transporter by preventing excess aggregation and coagulation [7]. The above standard PSC configuration has produced the most efficient cells; however, many researchers have questioned both the cost and the endurance of the organic components. In response to this concern, it has been suggested to completely eliminate the hole transporter and proceed with the construction of hole-transporter-free PSC devices. This choice is permitted by the fact that organometal halide perovskites are excellent hole transporters themselves, therefore they can function without the need of an additional organic hole transporter. This has indeed been verified and reported in several publications [11,12,13,14,15]. The efficiency of hole-transporter-free cells is lower than those which have an organic hole transporter, however, what one can gain in terms of cell cost and cell stability is equally or maybe more important than record efficiencies.

**Figure 1:**
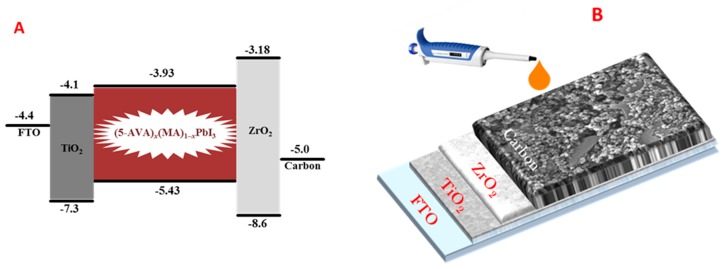
(**A**) Energy diagram showing the fitting of the energy levels between those of the perovskite and those of the charge collectors, i.e., titania for the electrons and carbon for the holes. (**B**) Arrangement of the mesoporous layers forming the device scaffold.

Carbonaceous materials provide a successful alternative for the construction of hole-transporter-free PSC variants [11,12,13,14,15]. A preferred configuration, which we have also adopted for the present work is described by the following scheme: FTO/bl-TiO_2_/m-TiO_2_/buffer layer/Carbon. Buffer and carbon layers consist of mesoporous materials through which the liquid perovskite precursors are infiltrated. Perovskite is then formed and disperses throughout the three mesoporous layers of titania, buffer, and carbon. As buffer, most researchers use zirconia, while carbon collects holes and at the same time plays the role of cell counter electrode. The carbon layer is composed of a mixture of graphite with carbon black (i.e., commercial carbon nanoparticles). This mixture is electrically conductive and energetically favorably located to scan holes in the perovskite valence band [16,17] (cf. Figure 1). Of course, other carbon layer compositions, including popular carbonaceous variants, such as carbon nanotubes or graphene, may be employed, but simple graphite-carbon black pastes [18,19] can offer excellent carbon electrodes. In the present work, we opted for the same PSC configuration, as summarized and pictorially represented by Figure 1.

There are a number of issues that we have chosen to deal with in the present work: (1) The presence of a carbon layer-electrode in a PSC discourages, thanks to its hydrophobicity, the entrance of water droplets, which are the worst enemy of organometal halide perovskites, since water decomposes them. It is presently verified that once perovskite was formed, the carbon layer provided a long term protection; (2) the addition of an organic hole transporter in a carbon electrode-based PSC brings an increase in efficiency. The importance of this increase is presently assessed in terms of photovoltaic parameters modification; (3) the choice of the titania-zirconia-carbon configuration allows the construction of devices by printing techniques, which may be easily up-scaled. Examples of cell construction by using screen printing are presently demonstrated. In this respect, the present work is a continuation of a previously published report [20].

## 2. Materials and Methods

Unless otherwise specified, all reagents were purchased from Sigma-Aldrich (St. Louis, MO, USA) and were used as received. Commercial nanocrystalline Titania Degussa P25 was used for the deposition of the mesoporous Titania layer. SnO_2_: F transparent conductive electrodes (FTO, Resistance 8 Ω/square) were purchased from Pilkington (Merseyside, England) while 5-ammonium valeric acid iodide (5-AVAI) was from Dyesol (New South Wales, Australia). 

### 2.1. Synthesis of Pastes for Screen Printing

The procedure for the synthesis of homemade pastes for printing titania, zirconia and carbon were the same as described in our previous publication [20], where a detailed characterization of the screens used for screen-printing is also given.

### 2.2. Device Construction

An FTO glass was etched with Zn powder and HCl (37%) to obtain two detached electrodes, and then it was ultrasonically cleaned successively with detergent, ethanol, and deionized water. A TiO_2_ compact layer was then deposited on FTO substrate by spin casting a colloidal (sol-gel) dispersion of titanium isopropoxide in absolute ethanol for 30 s at 3000 rpm. The colloidal dispersion was made by adding, drop by drop, 2.5 mL of a solution of 2M HCl in ethanol in a solution of 350 µL titanium isopropoxide in 2.5 mL of ethanol under stirring. When the dispersion became clear, it was ready for use. After drying the coated substrate at 100 °C for 10 min, it was sintered by calcination at 500 °C for 20 min. It must be noted at this point that spin-coating is, of course, not a unique method to make the compact titania layer. Spraying can be employed as well, in conformity with printing procedures. Spin-coating was just convenient in the present case. In a next step, a 400 nm thick mesoporous TiO_2_ layer made of P25 commercial powder was screen printed on the compact layer and dried at 100 °C for 10 min, followed by sintering at 500 °C for 20 min. Then a 1.7 μm thick mesoporous ZrO_2_ insolating layer was deposited on the top of the mesoporous TiO_2_ layer by screen-printing dried at 125 °C and sintered at 450 °C for 20 min. After cooling to room temperature, a 25 μm thick mesoporous carbon layer was printed on the top by screen printing and sintered at 400 °C for 30 min. Film thicknesses were measured by recording the FESEM images of a cross section of the cell. Finally, 4 μL of the perovskite precursor liquid (5-AVA)_x_(MA)_1−x_PbI_3_ was dropped on the top of the carbon electrode, where it infiltrated through the pores, and it was at the end annealed at 50 °C for 1 h. Device construction is pictorially represented by Figure 1B. It must be underlined that the above film thicknesses refer to the optimal values. Several other thickness combinations have been tested. Hole transporter spiro-OMeTAD was added on the top, when necessary, by using a solution of 36 mg/mL in chlorobenzene, which was spread on the top of the carbon layer (after the introduction of the perovskite) and was followed by spinning at 3000 rpm for 1 min and annealing at 60 °C for 5 min. Devices of several active surface areas were constructed. Small cells were 5 mm × 10 mm, while bigger cells were either 1 cm × 1.5 cm or 2 cm × 1.5 cm. Photovoltaic characteristics were mostly recorded by applying a mask of size 2 mm × 5 mm.

### 2.3. Characterization

Photocurrent density-voltage (J-V) curves were recorded by using a Keithley 2601 (Keithley Instruments, Solon, OH, USA) source meter and by illumination with a Solar Light simulator providing a beam of 100 mW cm^−2^ light intensity. All measurements on small cells were made by applying a mask with aperture size equal to 2 mm × 5 mm. Electrochemical Impedance Spectroscopy (EIS) characterization was carried out on PSC devices with a potentiostat/galvanostat (PGSTAT128N, Autolab B.V., The Netherlands) under both dark and AM-1.5G illuminated conditions.

## 3. Results and Discussion

As explained in the Experimental section, the devices studied in the present work were fabricated by first screen printing the consecutive layers of titania, zirconia, and carbon, and then infiltrating the perovskite precursor solution and any additives. The same fabrication route was followed both for small and for up-scaled devices.

**Figure 2:**
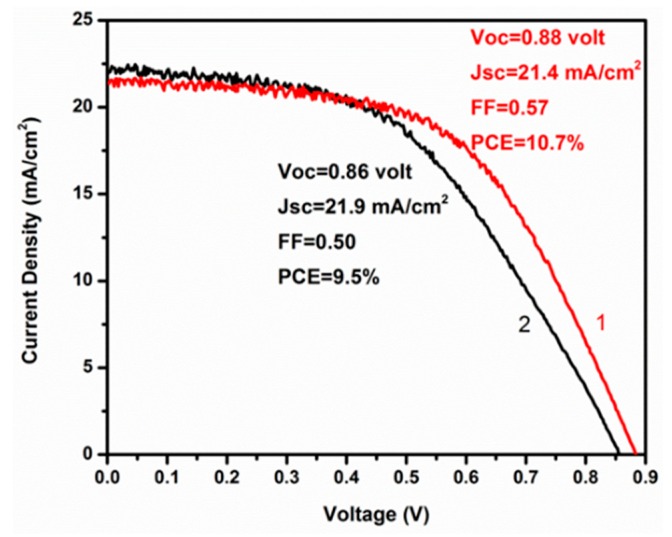
Density-voltage (J-V) plots for a freshly-made hole-transporter-free device (1) and for the same device stored for 180 days under ambient conditions (2).

All devices were constructed and characterized under ambient conditions. There are many authors that have recently attempted to assess PSC behavior under ambient conditions because this is an attractive situation that facilitates cell fabrication and decreases cost. It must be underlined that synthesis of perovskite under ambient conditions is only justified if ambient humidity is very low. Ambient humidity may vary day by day and month by month. In our case for devices made in Patras, Greece, it was okay to make cells on a dry summer day but this was not possible under late fall or winter conditions. In the latter case, it was necessary to manipulate perovskite synthesis in a dry box to assure low humidity (below 30%). Thus, a device made under ambient conditions of 50 to 60% relative humidity was 60% less efficient than a device made in a dry box of less than 30% relative humidity. Of course, the above result is expected in view of the known destructive effect that water has on organometal halide perovskites. The present information is added to eliminate any illusions on this matter. The crucial step is the introduction of perovskite by infiltration though the titania/zirconia/carbon stacks. Once the perovskite is formed, the carbon top layer discourages water penetration thanks to it hydrophobicity. We fabricated a cell in June and kept it under ambient conditions for 180 days. After this period of time, there was only a small loss of efficiency. Indeed, as seen in Figure 2 showing current density-voltage graphs for a fresh device and for the same device after 180 days, its stability was very satisfactory (only 11% drop in efficiency), even though during that period there was a progressive increase in average ambient humidity from about 30% to about 60%. This result clearly verifies the stabilizing effect that the carbon layer offers to these devices.

Nevertheless, the effect of humidity seems to be more intriguing than a simplified water destructive model. As explained in a recent work [21] where enhanced exposure of carbon based PSC devices to humidity was studied, such a forced exposure increased instead of decreasing cell efficiency. It was suggested that the presence of the carbon layer allows only gaseous water to pass through the mesoporous stack. This gaseous water assists perovskite crystal growth. As a consequence, perovskite becomes a more effective semiconductor. Water molecules can destroy a thin perovskite layer, but in the case of the thick layer formed in the mesoropous stack by precursor infiltration, gaseous water induces crystal growth [21]. In the absence of carbon, water droplets would have come in contact with the perovskite and destroy it anyway. Therefore, it seems to be very important to control humidity during the first stage of perovskite formation, i.e., during infiltration in the mesoporous stack, but once the perovskite is formed, the carbon layer provides an effective shelter.

**Figure 3:**
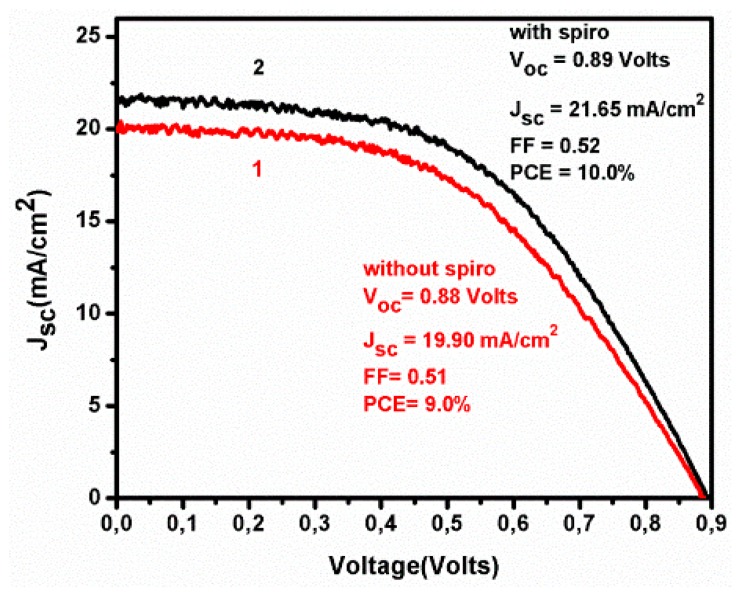
J-V plots for devices made without (1) and with spiro-OMeTAD (2).

The data of Figure 2 show that a device with a descent efficiency of PCE = 10.7% can be fabricated under ambient conditions by a simple procedure using fully printable (screen printed) components. The efficiencies reported by other researchers using similar carbon based PSC devices vary in a range that includes this value [13,20,21,22,23,24]. As already said, we have presently tested the possibility to increase device efficiency simply by adding an organic hole transporter. Plain spiro-OMeTAD was chosen for this purpose without any additives. spiro-OMeTAD was introduced by using its solution of 36 mg/mL in chlorobenzene, which was spread on the top of the carbon layer (after the introduction of the perovskite and the initial cell characterization) and then it was spun at 3000 rpm for 1 min and annealed at 60 °C for 5 min. As seen in Figure 3, the presence of this agent did increase device performance. All photovoltaic parameters, Jsc, Voc, and fill factor (FF) increased albeit to a small extent. 

**Figure 4:**
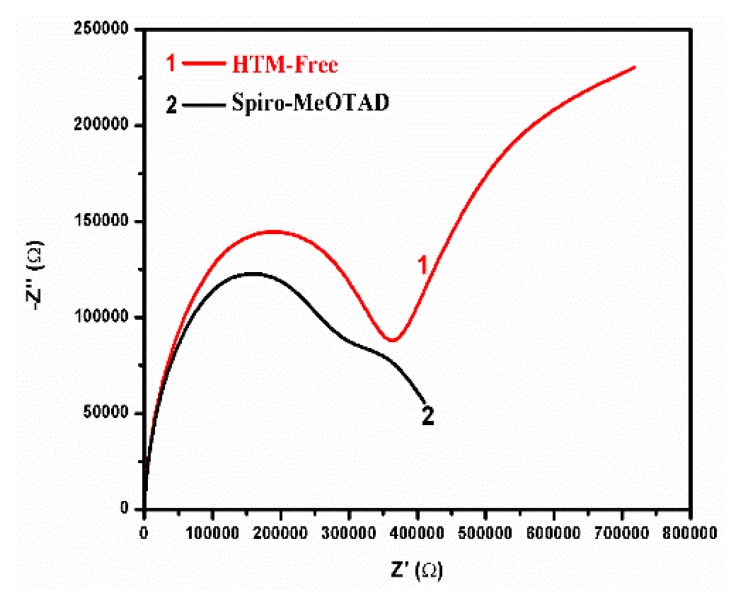
Nyquist plots for data obtained in the dark and by zero bias for devices constructed without (1) and with (2) spiro-OMeTAD.

In order to clarify the nature of the effect spiro has on device function, we have carried out Electrochemical Impedance Spectroscopy (EIS) characterization of devices made with and without the organic hole transporter. The data are listed in Table 1 while an example presenting Nyquist plots is given in Figure 4. Measurements were made under bias of 0.0 and 0.8 V. Best fitting was accomplished by circuit 1 in the case of 0.0 V and circuit 2 in the case of 0.8 V bias, respectively (circuits are illustrated in Table 1). The series resistance R_s_ underwent a small decrease in the presence of spiro under all experimental conditions. This shows that a net positive effect is produced on cell conductivity in the presence of the organic hole transporter. This may justify the higher current density obtained in its presence as shown in Figure 3. Resistance R_1_ is a component of both circuits (therefore, both biases). Because it fits the low frequency semicircle in the Nyquist plots of Figure 4, it is assigned to charge accumulation at the perovskite/charge collector interfaces and to charge recombination processes. The fitted values of R_1_ were relatively large in the dark but were dramatically decreased under illumination. In the dark, the presence of the organic hole transporter produced a net effect in decreasing resistance, however, under illumination the differences, if any, were not detected. This result enhances the indications that the influence of the hole transporter is of small importance. Nevertheless, R_2_ which corresponds to the high frequency semicircle in Figure 4 and is assigned to the charge transfer resistance at the perovskite/carbon interface, does indicate a net decrease of resistance in the presence of spiro at both dark and illumination conditions. In other words, there exists a beneficial effect produced in the presence of spiro but it is small. The remaining two resistances R_3_ and R_4_ appeared when a bias of 0.8V was applied (approximately equal to the Voc). Both showed an increase in the presence of spiro in the dark while under illumination their role, as well as any differences between devices containing or not containing spiro, were diminished. These characteristics fit resistances assigned to charge accumulation at the interfaces under strong bias. Apparently, such a charge accumulation loses its importance under illumination. The values then of R_3_ and R_4_ support the above proposed model. Same is true for the rest of the data from Table 1. In conclusion, the only net effect detected in the presence of spiro was a small decrease of the series resistance which justifies the data of Figure 3. Therefore, the presence of the organic hole transporter (alone without any additives) does increase cell performance but to a rather small extent. Then its complete elimination is a good trade against device construction cost. This, of course, does not mean that the presently used organic hole transporter or other hole transporters are of no use. It is simply a matter of further research.

**Figure 5:**
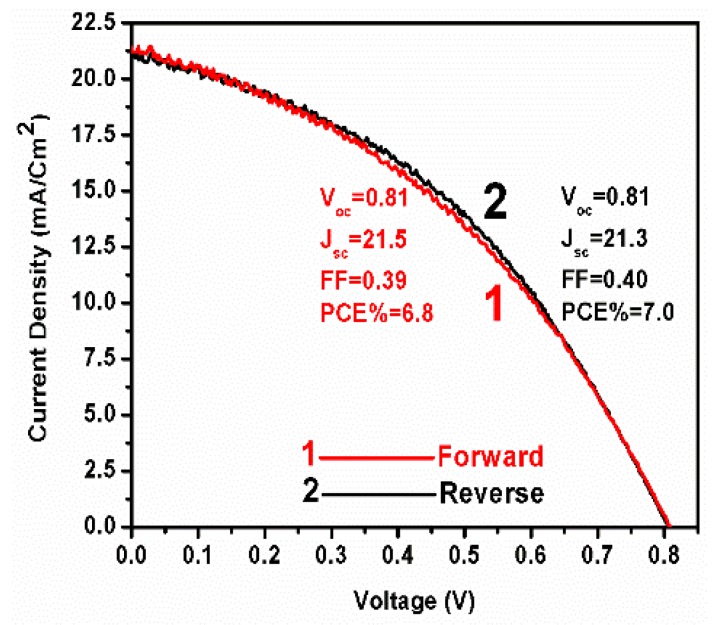
J-V plots for a 3 cm^2^ device under forward and reverse scan.

Up-scaling of the above small-size cells (size: 0.5 cm × 1.0 cm) was tested by construction of larger devices of either 1 cm × 1.5 cm or 2 cm × 1.5 cm. Variation of device dimension was easy, thanks to the employment of screen printing. Increase of device size had an adverse effect on the fill factor and, to a smaller extent, on device open-circuit voltage, as seen by the data of Figure 5. It was concluded that this result reflects device construction defects, which may increase shunt paths across the layers forming device scaffold. Obviously, device construction optimization by eliminating such deficiencies will be necessary. This matter is beyond the scope of the present work.

## 4. Conclusions

In the present work, we successfully constructed fully screen-printed hole-transporter-free perovskite solar cell devices with a decent maximum efficiency of 10.7%. The device was based on a titania-zirconia-carbon stack with perovskite formed by infiltration of its liquid precursors. It has been shown that addition of spiro-OMeTAD had a small favorable effect on device performance, mainly by increasing current. The results supported the protective effect that the carbon layer introduces for the preservation of the perovskite against humidity. Finally, increase of device size under the present conditions had an adverse effect on efficiency, obviously owing to defects introduced during layer printing. 

## Figures and Tables

**Table 1:**
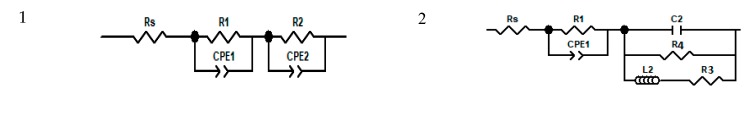
Electrochemical Impedance Spectroscopy (EIS) data obtained with devices made with or without an organic hole transporting material at various measurement conditions.

Addition of spiro-OMeTAD	Bias (V)	Light	Fitted circuit	R_s_^1^ (Ω)	R_1_^2^ (kΩ)	R_2_^3^ (kΩ)	R_3_^4^ (kΩ)	R_4_^4^ (kΩ)	CPE1-T^5^ (μF)	n_1_^6^	CPE2-T^7^ (nF)	n_2_^8^	C_2_^9^ (nF)
Yes	0.0	No	1	46	660	353	-	-	2.8	0.77	28.9	0.91	-
No	0.0	No	1	41	166	277	-	-	1	0.86	28.1	0.92	-
Yes	0.0	Yes	1	56	0.08	0.15	-	-	1.3 × 10^4^	0.38	1400	0.71	-
No	0.0	Yes	1	41	0.08	0.06	-	-	1.5 × 10^4^	0.51	3200	0.64	-
Yes	0.8	No	2	60	2.0	-	6.1	31	0.09	0.92	-	-	16
No	0.8	No	2	56	1.2	-	12.7	51	0.12	0.92	-	-	13
Yes	0.8	Yes	2	~0	0.09	-	~0	0.02	0.004	~1	-	-	6.8
No	0.8	Yes	2	~0	0.09	-	~0	0.02	0.007	~1	-	-	33

R_s_—Series Resistance R_1_—Charge accumulation and recombination resistance at the perovskite/charge collector interfaces R_2_—Charge transfer Resistance at Carbon/Perovskite interface R_3_ and R_4_—Charge accumulation resistances at the interfaces CPE1-T—Constant Phase Element formed at the perovskite charge collector interfaces n_1_—Power of CPE1 CPE2-T—Constant Phase Element formed at the Carbon/Perovskite interface n_2_—Power of CPE2 C_2_—Capacitor formed at the Carbon/Perovskite interface 3.3. Formatting of Mathematical Components.
